# Editorial: Responses to chronic stress in vertebrate animals: from molecules to behavior

**DOI:** 10.3389/fendo.2026.1875313

**Published:** 2026-05-22

**Authors:** Athanasios Samaras, Androniki Raftogianni, Marnix Gorissen

**Affiliations:** 1 Department of Biology, University of Crete, Heraklion, Greece; 2 Department of Psychology, Laboratory of Behavioral Neuroscience, University of Crete, Rethimo, Greece; 3 Department of Plant & Animal Biology, Radboud Institute for Biological and Environmental Sciences, Radboud University, Nijmegen, Netherlands

**Keywords:** cognition, cortisol, cortisol receptors, nanoplastics, neuroendocrine, serotonin, welfare

The stress response is a fundamentally adaptive biological function that enables organisms to cope with threats to homeostasis by triggering coordinated physiological and behavioral responses. However, when acute stressors become chronic, this adaptive machinery can turn maladaptive, compromising health, growth, reproduction, and cognitive function. Understanding chronic stress responses across multiple levels of biological organization is therefore a central challenge in comparative physiology, neuroendocrinology, and animal welfare science. This Research Topic was conceived to bridge gaps in the definition of chronic stress, evaluate its indicators, and examine vertebrate responses at both inter- and intra-specific levels.

The Research Topic comprises seven contributions that collectively advance our understanding of how chronic stress alters glucocorticoid dynamics and reshapes gene expression, metabolism, appetite regulation, brain monoaminergic signaling, immune function, and social behavior.

## Molecular HPI axis regulation under chronic stress

A key theme in this Research Topic is how glucocorticoid dynamics are regulated at the molecular level. Two original research articles directly address the tissue-specific regulation of the hypothalamus–pituitary–interrenal (HPI) axis in fish. Aravena-Canales et al. examined the molecular and epigenetic effects of crowding stress in rainbow trout (*Oncorhynchus mykiss*) skeletal muscle, a key tissue for growth and aquaculture productivity. By integrating RNA-seq with whole-genome bisulfite sequencing, they showed that chronic crowding elevates cortisol, induces oxidative DNA damage, and triggers epigenetic reprogramming. Pathway analyses identified autophagy, mitophagy, and insulin signaling as primary targets of these transcriptomic-epigenomic changes, with several genes showing inverse relationship between expression and methylation. This work represents one of the first integrative transcriptomic–methylomic profile of crowding stress in fish muscle and provides a framework for understanding long-term molecular imprints of aquaculture stressors on growth-related tissues.

Complementing this, Samaras et al. used a predictable repeated-stress protocol in European sea bass (*Dicentrarchus labrax*) to characterize HPI axis desensitization. Chronically stressed fish showed a blunted acute cortisol response and reduced circulating cortisone. Gene expression analyses across the brain, head kidney, liver, and gills revealed tissue-specific receptor remodeling. In the brain, chronic stress reduced *pomc* expression without compensatory changes in *mc2r*, indicating central dampening of the axis. In the head kidney, acute stress downregulated corticosteroid receptors (*gr1, gr2*, and *mr*), suggesting local desensitization of interrenal feedback. Together with maintained *hsd11b2*-mediated cortisol inactivation, these findings indicate progressive functional desensitization of the HPI axis without loss of basal homeostatic function.

## Receptor-specific cortisol signaling and metabolism

Metabolic responses to chronic cortisol depend on the receptors mediating the signal. Antomagesh and Vijayan addressed this using zebrafish (*Danio rerio*) and isotope tracing of ¹³C-glucose in glucocorticoid receptor (GR) and mineralocorticoid receptor (MR) knockout lines. Cortisol altered glucose flux across serum, liver, and brain, enhancing glycolysis and TCA cycle activity for energy production. However, receptor knockouts disrupted this metabolic reprogramming in a tissue-specific manner. GR and MR thus exert complementary control over energy allocation and metabolic homeostasis, jointly supporting stress adaptation. These findings highlight the conserved roles of glucocorticoids across vertebrates.

## Neuroendocrine control of appetite and serotonergic signaling

Two studies in Atlantic salmon (*Salmo salar*) illustrate how temporal context shapes neuroendocrine interpretation. Lai et al. exposed parr to 21 days of unpredictable chronic stress followed by an acute stressor. Chronic stress reduced feed intake and downregulated orexigenic *npya1* without engaging *crf1*, whereas acute stress upregulated both *crf1* and orexigenic neuropeptides, indicating a homeostatic rebound. These results demonstrate that single time-point measurements can misrepresent chronic physiological states and underscore the importance of temporal dynamics in appetite regulation.

Sørensen et al. studied serotonergic welfare indicators by studying fish across a welfare continuum in aquaculture sea cages. Region-specific quantification of serotonin (5-HT) and its metabolite 5-HIAA showed a functional dissociation: brainstem serotonergic activity reflected chronic welfare state, whereas telencephalic turnover reflected acute stress reactivity, but only in fish with adequate baseline welfare. Thus, serotonergic biomarkers are context-dependent and require region-specific, welfare-informed interpretation.

## Social stress, cognition, and immune function

Winberg and Hubená reviewed how social hierarchy formation generates long-term physiological and behavioral divergence in fish. Grounded in appraisal theory, they show that dominance interactions produce stable differences between dominant and subordinate individuals across HPI axis function, monoaminergic signaling, immune responses, and cognition. Subordinates, in particular, exhibit negative cognitive bias under ambiguous conditions. These patterns link stress coping styles (proactive vs. reactive) to both genetic and environmental influences. The authors also highlight that selecting for proactive traits in aquaculture may produce unintended welfare costs when individuals become socially subordinate.

## Multi-stressor ecotoxicology

Ruiz et al. placed stress physiology in an environmental context by examining gilthead seabream (*Sparus aurata*) exposed to elevated salinity and polystyrene nanoplastics. Both stressors independently disrupted immunoendocrine function across mucosal tissues (skin, gills, intestine), and combined exposure produced interactive, non-additive effects. These findings highlight the importance of multi-stressor designs in ecotoxicology and reinforce the relevance of chronic stress pathways for climate change research and aquaculture sustainability.

## Synthesis and future directions

Collectively, these studies demonstrate that chronic stress regulation is a multi-level process involving coordinated changes in endocrine signaling, gene regulation, metabolism, immune function, and behavior. Chronic stress reshapes HPI axis function via altered glucocorticoid dynamics, receptor expression, and epigenetic remodeling, while modifying appetite regulation and serotonergic signaling. At the organismal level, it drives long-term changes in growth, immunity, and social behavior, with environmental stressors engaging overlapping pathways.

Key questions remain regarding the long-term effects of early-life stress and heritability of stress-induced epigenetic changes. The GR/MR receptor-partitioning framework requires validation across vertebrates, particularly in mammals, where MR plays a more constricted role in corticosteroid signaling. In addition, the temporal dynamics of stress, from adaptation, to dysregulation, and recovery, remain largely unknown and require longitudinal approaches. Future research should link molecular mechanisms to organismal performance while accounting for individual variation, using multi-level datasets, and should employ ecologically realistic multi-stressor designs. Finally, given the link between chronic stress and welfare in farmed animals, robust longitudinal biomarkers combining physiological, neuroendocrine, and behavioral measures should be validated against long-term welfare and production outcomes in commercial aquaculture.

